# State Work with Infectious Diseases of Animals1Presented at the annual meeting of the State Agricultural Society, at St. Paul, January 10 and 11, 1900.

**Published:** 1900-06

**Authors:** M. H. Reynolds

**Affiliations:** St. Anthony Park, Minn.


### STATE WORK WITH INFECTIOUS DISEASES OF ANIMALS.[1]

By M. H. Reynolds, M.D., V.M.,
ST. ANTHONY PARK, MINN.

(Concluded from page 273.)

*What Other Public Authorities Are Doing.* Pennsylvania tests cattle at the expense of the State, furnishing veterinarian and tuberculin. State pays partial valuation for condemned cattle. State Veterinarian reports that when this work was commenced three years ago the task seemed almost hopeless. Statistics show constant diminution of reacting cattle, and the inference seems reasonable that tuberculosis is less prevalent in that State than when the work began. Up to June 1, 1897, the average percentage had been 20.4. From this time until January, 1898, 13.73 per cent. During 1898, 14,437 cattle were tested in this way ; 1348 reacted —12.9 per cent.

Pennsylvania tests only upon application, and not only that but application must show reasonable evidence that tuberculosis exists in the herd. Their annual report indicates that applications are much more numerous than can be granted. The owner is required to submit reasons for making the examination. He must make application for test and agree to do all in his power to keep the herd free from tuberculosis in the future. Quite a different situation from what we have here in the West.

Pennsylvania prohibits the importation of dairy or breeding cattle except upon tuberculin-test. No compensation is allowed for cattle imported contrary to law.

Massachusetts is now appropriating about $75,000 annually for the work with infectious diseases, a very large proportion of which is used in the work with bovine tuberculosis. Pennsylvania has an annual appropriation of about $40,000 for dealing with infectious diseases of domestic animals. Of this appropriation a very large portion—about $28,000—is used for dealing with bovine tuberculosis.

Illinois has recently put into effect an order prohibiting the importation of untested cattle for breeding or dairy purposes. Iowa is making quite an active stir concerning this problem, although she may not take any radical step at this time. Yesterday a tuber-

[1] Presented at the annual meeting of the State Agricultural Society, at St. Paul, January 10 and 11, 1900.

culosis convention was called to order in Des Moines for the express purpose of discussing the problems connected with bovine tuberculosis and to recommend such measures as seem wise. Prominent speakers from various parts of the United States were on the programme. The State Veterinary Association of Iowa is in session to-day and to-morrow, and it is the expressed purpose of this Association to bring influence to bear upon the Legislature looking toward legislation similar to that in effect in other States.[1]

In view of recent developments in certain of the Eastern States and among our neighboring States here in the West, it may possibly become necessary for Minnesota to take some radical action concerning the importation of dairy and breeding cattle. I have hitherto taken the position, so far as Minnesota was concerned, that so long as cattle in neighboring States were probably no worse affected with tuberculosis than our own we were not justified in prohibiting the importation of untested cattle. This proposition came up in Wisconsin something like a year ago, and I made a similar proposition to the Wisconsin representatives that as long as Wisconsin was no better in this respect than her neighboring States there would be nothing gained by such a measure. But this problem has assumed a different phase. When our neighboring States adopt measures similar to those now in force in Illinois, Minnesota will be compelled to do likewise or become a dumping-ground for tuberculous cattle. A considerable number of cattle, largely dairy cows, are shipped each year from this State to Illinois. Nearly all of these cattle are tested before shipment. The healthy cattle are shipped into Illinois, the tuberculous cattle are left on our hands. If dairy or breeding cattle are tested in Iowa with a view to shipping into Illinois, the same thing occurs ; the healthy cattle are shipped into Illinois, the diseased cattle may be moved over the line into Minnesota.

The following table is taken from our Experiment Station *Bulletin*, No. 51, and is quite suggestive as to the influence concerning the prevalence of this disease among natives, high-grades, pure breds, city dairies, and in the various conditions of stables :

[1] Since this paper was written Missouri has issued a proclamation similar to that now in force in Illinois.

TABLE XLVIII.—PREVALENCE ACCORDING TO CLASS AND CONDITION.

Table Summary: Greatest prevalence among "pure breds," "city dairies," "poor condition of stable," and "poor ventilation." Farm conditions—good stables and ventilation do not protect infection, however.[1]

| No. | Class. | No of herds tested. | No. of animals tested. | No of re-actions. | Per cent. tuber-culous. |
|---|---|---|---|---|---|
| 1 | Natives . . . . . . | 137 | 2839 | 223 | 7.8 |
| 2 | High-grades . . . . . | 5 | 157 | 17 | 10.8 |
| 3 | Pure breds . . . . . . | 6 | 258 | 43 | 16.6 |
| 4 | Farm herds . . . . . . | 38 | 694 | 99 | *14.2 (7.8) |
| 5 | City dairy herds . . . . | 108 | 2736 | 284 | 10.4 |
| 6 | In "good" general condition of stable . . . . . . . | 57 | 1370 | 139 | *10 1 (6.8) |
| 7 | In "fair" condition of stables . | 59 | 1140 | 83 | 7.28 |
| 8 | In "poor" condition of stables . | 32 | 864 | 165 | 19.1 |
| 9 | With "good" ventilation . . | 45 | 1011 | 90 | *9.8 (5) |
| 10 | With "fair" ventilation . . | 45 | 1087 | 67 | 6.16 |
| 11 | With "poor" ventilation . . . | 48 | 1210 | 201 | 16.6 |

This data was collected in the most impartial way that could be devised, and with the sole view of investigation. This showing is what any student of sanitary matters should expect. The mere fact that the percentage of tuberculosis is highest in pure-bred herds should not suggest that men should not breed pure-bred cattle. It only suggests that men should not establish herds upon tuberculous foundation. They should breed healthy pure-bred cattle, not tuberculous pure-bred cattle. The figures here given should be taken as percentages for certain classes, and not as representing the entire cattle stock of Minnesota.

During the past three months there have been tested in this State 2768 cattle of all kinds, of which 135 reacted and 25 were killed; an average of 5.1 reactions. But the majority of these were not breeding herds. All of the dairy and breeding herds belonging to State institutions in Minnesota are now being tested regularly, and the officers of these various institutions are now in sympathy with this movement, although it was rather difficult to get the work started.

A careful experiment which was conducted on a rather large scale by the Experiment Station several years ago demonstrated quite fully that the tuberculin test was practically without effect upon dairy cows in full flow of milk, or upon fattening steers,

[1] Fifty-five of these tuberculous animals in each case—(groups 4, 6, and *9) were from the same two herds. Eliminating these two herds from the groups 4, 6, and 9, the percentages are reduced respectively to 7.8, 6.8, and 5.

and the effect upon tuberculous cattle was favorable rather than otherwise.

I wish to suggest a conference committee, consisting of five representative breeders of the State Agricultural Society and Stock Breeders' Association, to confer with representatives of the State Board of Health and Experiment Station. Let representatives of these two societies be experienced breeders, men who are personally and financially interested in a proper and safe solution of the problem.

What will Minnesota do? What is the wish of the stockmen of Minnesota concerning this great problem? Is it worth our serious consideration? What will we do about it?

